# RNA-binding proteins as a molecular link between COPD and pulmonary hypertension

**DOI:** 10.7150/ijms.108587

**Published:** 2025-03-29

**Authors:** Yi Liu, Ran Wang, Tao Jiang

**Affiliations:** 1 Department of Respiratory and Critical Care Medicine, the Fourth Affiliated Hospital of School of Medicine, and International School of Medicine, International Institutes of Medicine, Zhejiang University, Yiwu 322000, China.; 2 Department of respiratory and critical care medicine, the First Affiliated Hospital of Anhui Medical University, 210 Jixi Road, Hefei, Anhui 230022, China.

**Keywords:** RBPs, COPD, PH, posttranscriptional gene regulation, pulmonary vascular resistance, pulmonary vascular remodeling

## Abstract

Pulmonary hypertension (PH) is a vascular disease characterized by remodeling of the pulmonary arteries and right heart failure. Chronic obstructive pulmonary disease (COPD) patients often have PH, which can worsen symptoms and raise morbidity and mortality. There are several reasons for increased pulmonary vascular resistance, pulmonary vascular remodeling, and ultimately the development of PH in COPD. These factors include genetics, inflammation caused by chemicals breathed, and changes in the alveoli seen in COPD and its physiology. Genes involved in mRNA conversion, subcellular localization, splicing, and translation are all finely tuned by RBPs in their post-transcriptional regulation. Erythropoietin regulates cytokines, chemokines, proteins, growth factors, and other pro-inflammatory mediators that change the lung microenvironment. Over the past few years, we have learned more about how RBPs act in PH and COPD. Here, we discuss the existing understanding of RBPs' location in the same pathogenic pathways shared by PH and COPD in order to emphasize their potential relevance as disease determinant/biomarker and, consequently, for possible therapeutic targeting.

## Introduction

Both pulmonary hypertension (PH) and chronic obstructive pulmonary disease (COPD) are crippling conditions that have a major global influence on morbidity and mortality. Airflow restriction and ongoing respiratory symptoms are hallmarks of chronic obstructive pulmonary disease (COPD), which is often progressive and not entirely reversible^1^. Long-term exposure to dangerous particles or gases, most frequently cigarette smoke, is the main cause of the illness, which results in emphysema, chronic inflammation, and airway remodeling^2^, ^3^. Conversely, PH is a vascular disease that causes right heart failure and mortality due to elevated pulmonary artery pressure and pulmonary vascular resistance^4^, ^5^. A complex process comprising genetic alterations, inflammation, and metabolic problems contributes to the pathophysiology of PH^6^-^10^.

The role of RNA-binding proteins (RBPs) in the pathophysiology of PH and COPD is one of the main connections between the two conditions. A varied class of proteins known as RBPs are essential for post-transcriptional control of gene expression, including translation, stability, and mRNA splicing^11^-^18^. Numerous illnesses, including as cancer, heart disease, and respiratory disorders, have been linked to RBP dysregulation^19^-^25^. RBPs regulate important processes as inflammation, vascular remodeling, and metabolic reprogramming in COPD and PH^26^-^35^. The growing significance of RBPs in the pathophysiology of PH and COPD has been brought to light by recent research. For instance, it has been demonstrated that the RBP HuR contributes to chronic inflammation in COPD by stabilizing the mRNAs of pro-inflammatory cytokines such TNF-α and IL-1β^36^. HuR has been linked to the control of soluble guanylate cyclase (sGC) expression in PH, which is essential for vascular remodeling and vasodilation^37^. Furthermore, it has been demonstrated that additional RBPs, including SFPQ and PTBP1, control the transcription and splicing of genes related to metabolic reprogramming and vascular remodeling in PH^37^-^40^. Understanding the precise functions and methods of action of RBPs may help provide new insights into the pathophysiology of COPD and PH and suggest possible treatment targets, especially in light of the mounting evidence of their involvement in these conditions. The purpose of this study is to provide an overview of the current understanding of RBPs' involvement in PH and COPD, with an emphasis on their developing function as molecular bridges connecting the two disorders. This study advances our understanding by summarizing the existing knowledge of RBPs' involvement in COPD and PH, highlighting their potential relevance as disease determinants/biomarkers and therapeutic targets. It also discusses the interplay between RBPs and ncRNAs in the pathophysiology of COPD and PH, emphasizing mutual regulation mechanisms. Additionally, the study analyzes specific RBP inhibitors and their therapeutic implications in COPD and PH contexts, including their feasibility and potential side effects. Finally, it proposes potential experimental designs or hypotheses to explore unexplored RBPs or their modifications under COPD-PH comorbidity.

## Molecular factors that connect PH and COPD

**Figure 1:**
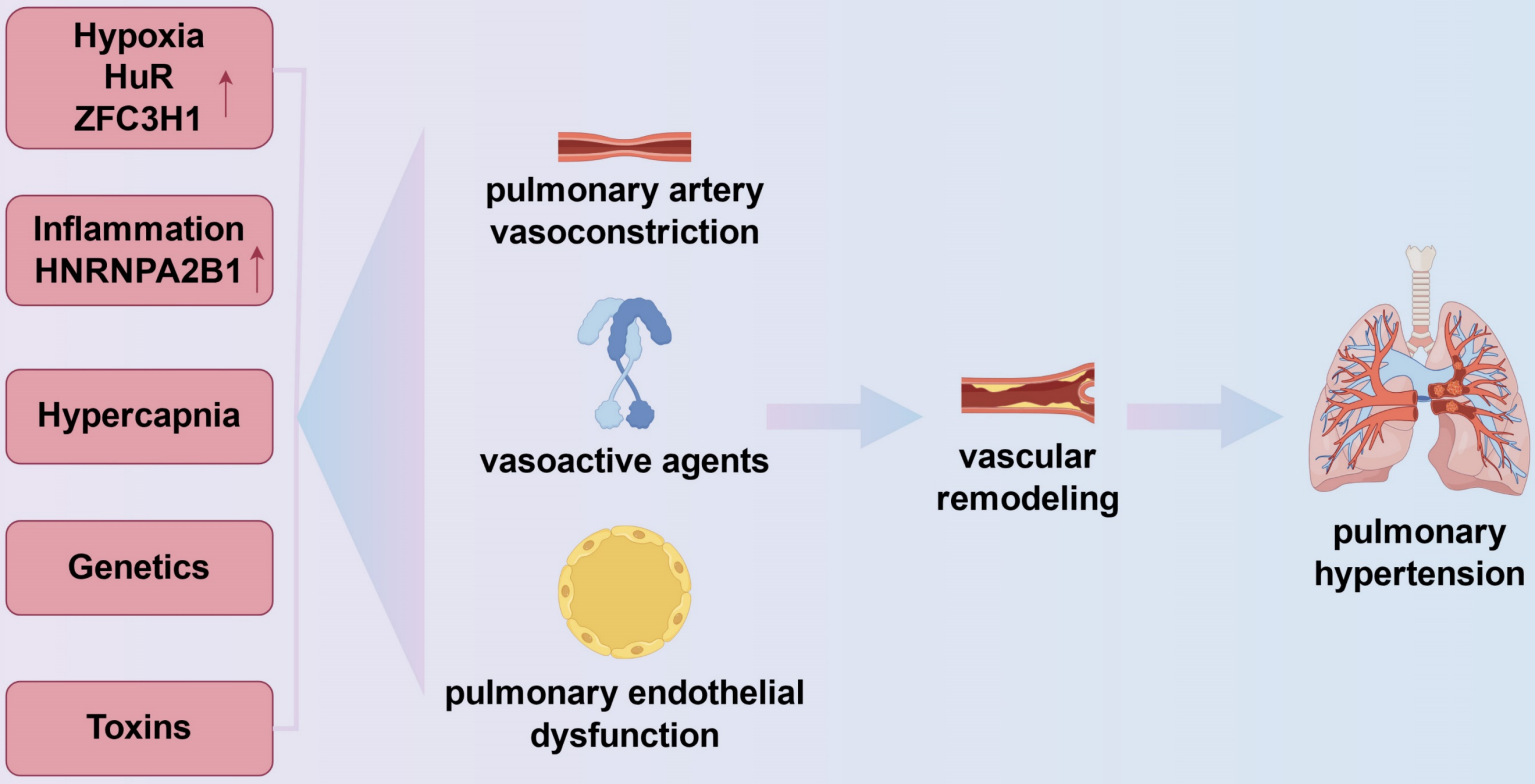
Mechanisms of pulmonary hypertension in chronic obstructive pulmonary disease. The onset of pulmonary hypertension in chronic obstructive pulmonary disease is linked to vasoconstriction, vascular remodeling, and the expression of vasoactive substances like vascular endothelial growth factor and endothelin-1, which are influenced by inflammation, genetics, hypoxia, and hypercapnia. In the pulmonary vasculature, hypoxia can exacerbate inflammation by upregulating the expression of HuR, an RBP that stabilizes mRNAs encoding pro-inflammatory cytokines including TNF-α and IL-1β. Furthermore, ZFC3H1 expression can be upregulated in hypoxia to mediate the repression of BRD4 and HIF1α RNAs. Chronic hypoxia also results in pulmonary hypertension by prolonged pulmonary vasoconstriction. Cell cycle checkpoints in pulmonary artery smooth muscle cells are regulated by inflammation-related RBPs (e.g., HNRNPA2B1).

Increased PVR, pulmonary vascular remodeling, and eventually the onset of PH is caused by a number of causes in COPD. These determinants include heredity, inflammation from inhaled chemicals, and alveolar alterations seen in COPD and COPD physiology (Fig.1).

The most significant contributing cause to an elevated PVR is most likely alveolar hypoxia. Acute hypoxia causes an increase in PVR in humans and nearly all animal species, which is explained by hypoxic pulmonary vasoconstriction^41^. More recently, the exact mechanism has been determined. The pulmonary smooth muscle cells' potassium calcium channels imply that when hypoxia occurs, potassium channels shut, causing membrane depolarization and calcium influx, which results in muscle contraction^42^. Blood viscosity increases as a result of hypoxic secondary polycythemia, which has also been linked to elevated PVR^43^. *In vitro* research and animal models of hypoxia-induced PH are the primary sources of information about the long-term impact of hypoxia on the pulmonary circulation^44^. All of the pulmonary vascular wall's cells and extracellular matrix have altered in both form and function. For example, the acute effect of hypoxia on voltage-dependent potassium channels is amplified and prolonged by chronic hypoxia, which enhances the production and release of endothelin from endothelial cells^45^. Hypoxia can upregulate the expression of HuR, an RBP that stabilizes mRNAs encoding pro-inflammatory cytokines such as TNF-α and IL-1β, thereby exacerbating inflammation in the pulmonary vasculature^37^. Besides, the inhibition of HIF1α and BRD4 RNAs can be mediated by hypoxia, which can also increase the production of ZFC3H1, a Celastramycin binding partner that regulates nuclear RNA stability. Chronic hypoxia also contributes to pulmonary hypertension by creating persistent pulmonary vasoconstriction^46^. Chronic hypoxia affects two powerful vasodilators produced by endothelial cells: prostacyclin and nitric oxide. This results in a proliferative condition of vascular wall cells and an imbalance favoring a rise in pulmonary vascular tone.

Inflammatory cells inside the vascular wall have been implicated in the development of structural and functional problems of the pulmonary arteries (PA) in COPD. When Joppa et al. and his colleagues compared inflammatory markers in patients with and without PH who had COPD, they found that patients with pulmonary hypertension had greater levels of TNF-α and C-reactive protein^47^. Other groups discovered a correlation between cigarette smoking and decreased eNOS expression in PA. The changes in the endothelial function and pulmonary artery structure in cigarette-smoke-induced respiratory disease may be attributed in part to the decreased generation of nitric oxide^48^. In order to investigate whether smoking-related inflammatory processes could contribute to the development of pulmonary vascular abnormalities in COPD, Peinado et al. described inflammatory cell infiltration and endothelial dependent dilatation in the pulmonary artery (PA) of 39 patients undergoing pulmonary resection^49^. They came to the conclusion that smoking cigarettes causes a CD8(+) T-lymphocyte infiltrate in PA, which is linked to the impairment of the vessel's structure and function. This suggests that an inflammatory process may be involved in the pathogenesis of pulmonary vascular abnormalities in the early stages of COPD. Additionally, it has been seen in animal studies that exposure to tobacco smoke increases the gene expression of vasoactive molecules such endothelin-1 and vascular endothelial growth factor, which most likely adds to the combination of variables that cause high pulmonary artery pressures in COPD^50^. Inflammation-related RBPs (e.g., HNRNPA2B1) regulate cell cycle checkpoints in PH-pulmonary artery smooth muscle cells (PASMCs)^51^.

Both the pulmonary vasculature and right ventricular function may be impacted by hypercarbia. Hypercapnia and its related acidosis have been demonstrated in animal models to enhance PVR and induce poor right ventricular function^52^, ^53^. The impact of prolonged hypercapnia on PH in COPD is not well studied in large prospective studies. A right cardiac catheterization and blood gas measurements were recently used to evaluate 225 patients with chronic pulmonary illnesses. Both SaO_2_ and PaCO_2_ were found to be independent variables of mean pulmonary arterial pressure by multiple regression analysis. In light of the poor prognosis associated with borderline PH, removing excess pulmonary carbon dioxide in hypercapnia may be a significant therapeutic approach for chronic pulmonary disease^54^.

Numerous genetic variations have been linked to PH in COPD and pulmonary vascular disease. Increased ACE levels and the development of PH have been linked to polymorphisms in the angiotensin-converting enzyme (ACE) gene^55^. COPD patients may have eNOS gene polymorphisms, which might result in PH development^56^. Finally, it has been observed that vascular remodeling and hyperplasia caused by variation in the serotonin transporter 5-HTT gene result in PH^57^. Genetic polymorphisms in RBPs, such as single nucleotide polymorphisms (SNPs), have been associated with altered RBP function and disease susceptibility^58^. Additionally, epigenetic modifications, such as DNA methylation and histone modification, can regulate the expression and activity of RBPs. For example, the expression of HuR can be modulated by DNA methylation, and this may contribute to its dysregulation in COPD and PH^59^. The knowledge of genetic and epigenetic modifications of RBPs could be used toward translational research aimed at modelling disease processes and developing specific interventional approaches. For instance, studies could investigate whether genetic polymorphisms or epigenetic modifications of RBPs could serve as biomarkers for disease risk or therapeutic response. Additionally, epigenetic therapies targeting RBPs, such as DNA methyltransferase inhibitors or histone deacetylase inhibitors, could be developed to modulate RBP activity and improve disease outcomes.

## An overview of the structure and pathogenic roles of RNA-binding proteins

The maturation, stability, transport, and destruction of cellular RNAs are all regulated by RBPs, a diverse collection of PTGR modulators^60^-^70^. By attaching to conserved sequences mostly found in the untranslated regions (UTRs) of specific mRNAs, RBPs primarily function as a component of ribonucleoprotein (RNP) complexes^27^. RBPs' functional activity is defined by their modular structure, which is made up of the repetition of many domains^71^. Canonical RNA-binding domains (RBDs), including zinc-finger domain, RNA recognition motif (RRM), K homology (KH) domain, DEAD motif, and double-stranded RNA-binding motif (DSRM), are present in most RBPs^27^. Additionally, the co-existence of several binding domains and the beginning of chemical interactions (hydrogen bonds, stacking interactions, weaker interactions) improve the specificity of RBPs-RNA connection^72^. Furthermore, some highly conserved "unconventional" RBPs missing the standard RBDs were discovered by recent proteome-wide techniques. By use of unfolded and flexible protein regions that contain repeating motifs rich in glycine, lysine, and arginine-known as intrinsically disordered regions (IDRs)-they facilitate both highly selective and nonspecific RNA binding^73^. Nonconventional RBP-mediated interactions encourage protein-RNA co-folding, shape-complementary association, protein-RNA complex scaffolding, or modification of the bound protein's function. TRIM25, an ISG15 ligase, the E3 ubiquitin, regulators of alternative splicing, metabolic enzymes like 3-hydroxyacyl-CoA dehydrogenase type 2 (HSD17B10), and others are examples of unconventional RBPs. The dynamic interaction between RBP and RBP further complicates the regulation mechanisms of shared target mRNAs. In particular, the cooperative model is the synergistic interaction of two RBPs with the same regulatory purpose, as opposed to the competitive model, where the antagonistic interaction results in a distinct regulatory outcome^74^.

**Figure 2:**
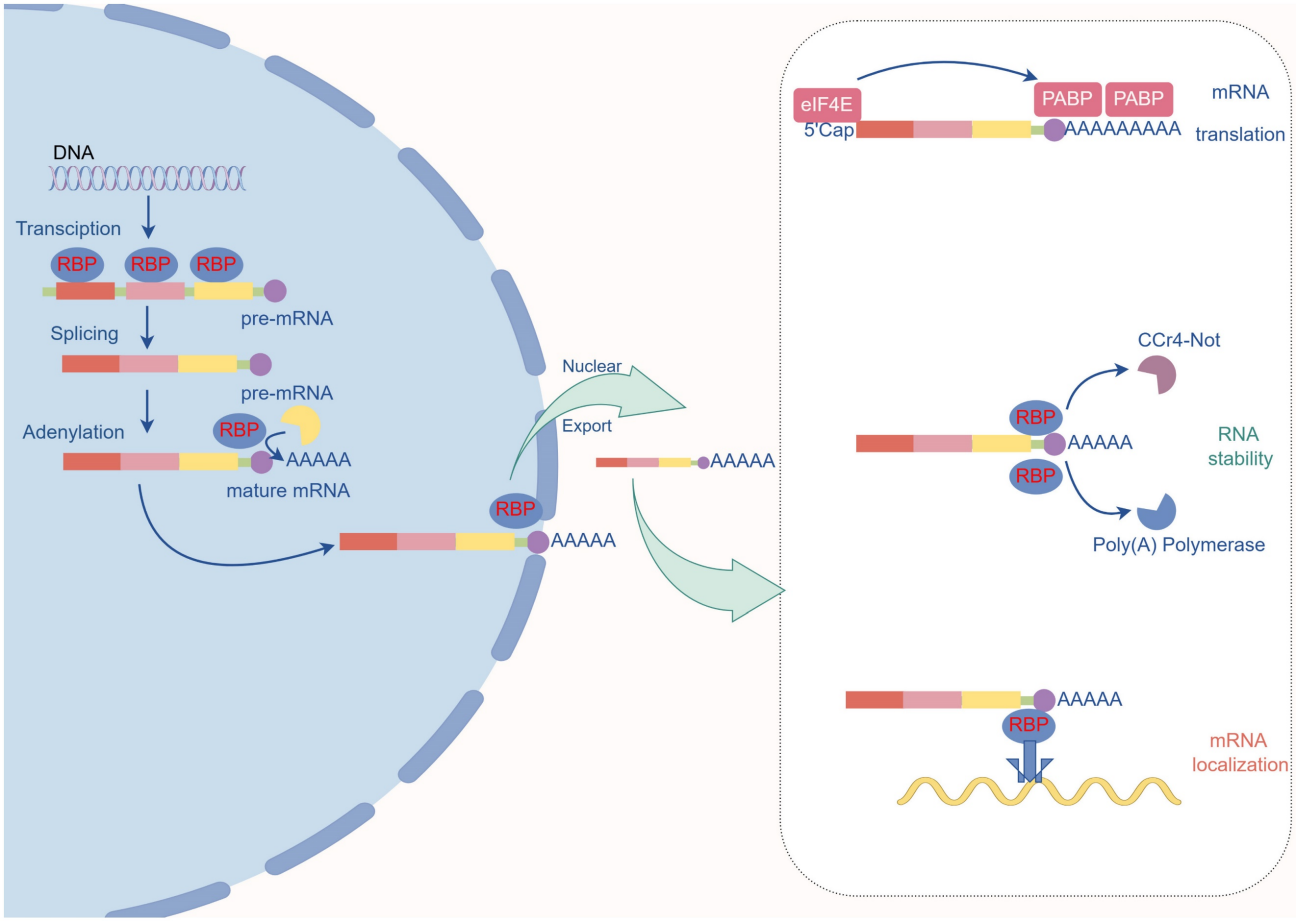
**The primary role of RNA-binding proteins. Role in Pre-mRNA Maturation:** RBPs bind to specific sites on pre-mRNA in the nucleus to attract the spliceosome, which removes introns. **Poly(A) Tail Addition:** RBPs identify the poly(A) site and recruit poly(A) polymerase to add the poly(A) tail. **Nuclear Export of mRNA:** The RBP complex binds to the nuclear pore and mature mRNA, facilitating its export from the nucleus to the cytoplasm. **Protection and Translation Efficiency in the Cytoplasm:** Poly(A) binding protein (PABP) protects the poly(A) tail from exonucleases and interacts with eIF4E at the 5' cap to circularize the mRNA, enhancing translation efficiency. **mRNA Transport:** Certain RBP complexes recruit dyneins to transport mRNA to specific locations. **mRNA Degradation:** RBPs recruit the Ccr4-Not deadenylase complex to initiate the degradation process.

All facets of the mRNA life cycle, including splicing, expression, localization, and stability, are regulated by RNA-binding proteins (Fig.2).

An essential pre-mRNA processing step that is tightly regulated by RBPs is RNA splicing. Pre-mRNA splicing is carried out by RNA and protein complexes called splicers. Spliceosomes are formed by five small nuclear ribonucleoprotein particles (U1, U2, U4, U5, and U6 snRNPs). Splicers identify and bind to short consensus sequences around exon intron junctions, referred to as 50- (GU) and 30- (AG) splice sites, using microRNAs and proteins present in snRNPs. They then stimulate the joining of exons and the elimination of introns. Constitutive or universal precursor mRNA splicing is the term we use to describe this process^75^. Numerous splicing accessory proteins interact with RNA and/or proteins inside snRNP to enhance or decrease the binding of snRNP to 50 and/or 30 splicing sites, as well as to regulate conformational changes during splicing assembly and catalysis. Usually, these proteins accomplish this without attaching to pre-mRNA. Since the 50 and 30% splicing sites in the majority of eukaryotic pre-mRNA are degenerate, extra cis acting RNA sequence elements and trans acting RBPs can regulate the recruitment of spliceosomes and their binding to these sites^76^, ^77^. These extra cis acting RNA sequence elements (splicing enhancers and silencers, found in the pre-mRNA's introns and/or exons) interact with trans acting RBPs (splicing regulators: splicing activators bind to splicing enhancers, while splicing inhibitors bind to splicing silencers) to produce alternative splicing (AS). The most well-known splicing regulators are those belonging to the heteroribonucleoprotein (hnRNP) and serine/arginine rich protein (SR protein) families. Because they bind to splice silencing codons and promote exon skipping, HnRNPs are referred to be repressors. However, the SR protein, often referred to as an activator, attaches itself to splicing enhancers in order to encourage exon inclusion^78^. Each gene may encode many mRNA variants due to AS, some of which may not encode proteins or may have opposing or differing roles. AS regulatory factors, such as SR proteins and hnRNPs, are essential for physiology and illness, and AS is therefore one of the primary mechanisms for pathogenic and developmental control of cellular activity.

Prior to export and cytoplasmic translation, mRNA undergoes nuclear maturation by polyadenylation, which is the insertion of a poly(A) tail in 3′. Poly(A) binding protein (PABP) covers and shields the tail from deterioration. In order to boost translation efficiency, PABP additionally interacts with eIF4E attached to the 5′-Cap to circularize the mRNA. Post-termination ribosome recycling for translation reinitiation is encouraged by circularization. Thus, a very effective way to control mRNA translation is by cytoplasmic regulation of the poly(A) tail's length^79^.

In order to create proteins, certain mRNAs must first undergo transcription in the nucleus before being sent into the cytoplasm and translated. On the other hand, certain mRNA transcripts specifically target particular areas for local translation or distribution, which causes cytoplasmic proteins to be distributed asymmetrically^80^. By forming a secondary structure as a binding site for RBP, the particular binding element (postal code) in the target gene 3'UTR controls cellular localization, which in turn mediates RNA localization to particular subcellular compartments. RBP recognition of cis motifs is the primary mechanism by which RNA is localized subcellularly^81^. mRNAs can be located by a variety of methods, including passive diffusion, active transport, and protection against destruction at a particular locus. The effectiveness of passive diffusion is limited to tiny cells. Therefore, this process is only necessary for bacteria^82^. RBPs provide both active transport and mRNA protection. RBPs carry out active transport by identifying a certain motif. After binding to mRNAs, RBPs transport the mRNAs along the myosin chain by enlisting motor proteins^83^. The precise location of an RBP serves as the foundation for the mRNA protection mechanism. The mRNA will be bound by the RBP in its location, shielded from degradation, and translated. Rapid mRNA degradation will occur at any other site if the protective RBP is absent^84^.

The nucleus produces mRNA, which is then carried to the cytoplasm. To increase mRNA output, some mRNA output receptors, or RBPs, attach to RNA and engage with nuclear pore complexes. Once in the cytoplasm, mRNAs have several outcomes. mRNA's capacity to attach to ribosomes for protein synthesis, storage in inclusion bodies for later usage, or targeted destruction is likewise regulated by RBPs^85^. Adenosine/uridine rich regions, sometimes referred to as AU rich elements (AREs) are some of the most conservative and distinctive RBP binding sequences that are found in genes implicated in immunological response, inflammation, and carcinogenesis^86^, ^87^. In the context of pulmonary disorders, these genes have a crucial role in controlling PTGR. Others, like triphenylproline (TTP), AU rich element binding factor 1 (AUF-1), and KH type splicing regulatory protein (KSRP), are linked to the ARE sequences of the transcripts involved in these processes, mediating mRNA instability, while other RBPs, like human antigen R (HuR), have stabilizing effects.

## RBP Functions in PH and COPD

Since RBPs regulate the process of RNA metabolism and gene expression, they are becoming more and more acknowledged as significant actors in PH and COPD^58^, ^88^, ^89^. Through their effects on RNA metabolism, including transcription, RNA splicing, and stability, studies have clarified the function of RBPs in PH and COPD over the last ten years (Fig.1 and Table 1).

Research on RBPs' function in regulating gene transcription in PH is only being started. Reduced RBP splicing factor proline and glutamine-rich protein (SFPQ) levels have been shown to activate CD40 transcription in PH, which in turn activates pulmonary artery adventitial fibroblasts^38^. SFPQ may inhibit CD40 transcription by converting histone H3 tri-methylation at lysine 4 (H3K36me3) to H3K36ac modifications on its promoter through interaction with histone deacetylase-1 (HDAC1). CD40L-CD40 signaling promotes activation of pulmonary adventitial fibroblasts by increasing proliferation, migration, and pro-inflammatory activity of adventitial fibroblasts^38^.

Splicing factors have been shown in several studies to have a crucial role in controlling the splicing of mRNAs that are functionally significant in PH pathophysiology^90^. The etiology of this illness is attributed to metabolic reprogramming, according to the recently proposed "metabolic theory" of PH. Outer membrane fibroblasts isolated from hypertensive pulmonary artery walls (PH Fibs) in humans and cows exhibit constitutive reprogramming of glycolysis and mitochondrial metabolism, pro-inflammatory effects, and anti-apoptotic properties^39^. Researchers demonstrated that PH-Fibs produced much more PKM2 transcripts than the normal participants did using a human pyruvate kinase muscle (PKM) splicing reporter that comprises exons 8 to 11 of the human PKM gene. Even better, using siRNA technology to silence the polypyrimidine pathway binding protein 1 (PTBP1) dramatically decreased the transcription level of PKM2 in PH Fibs, confirming PTBP1's function in regulating the PKM-AS process in PH Fibs^90^. The most frequent cause of heritable pulmonary arterial hypertension (HPAH) is mutations in the bone morphogenic protein receptor 2 (BMPR2) gene. Cogan and colleagues shown that the HPAH penetrance of carriers of BMRP2 heterozygous germline mutations is significantly influenced by AS of BMPR2^91^. The normal BMRP2 allele's BMPR2 pre-mRNA will splice in a way that produces more isoform-B (without exon 12) transcript than isoform-A (containing all 13 exons) in mutation carriers with decreased production of serine arginine splicing factor 2 (SRSF2). The risk of developing PAH is higher for those who carry certain mutations. Therefore, during genetic counseling, BMRP2 heterozygous germline mutation carriers may benefit from additional information provided by measuring SRSF2 protein expression. Eleven PH patients from the first group and eleven age- and gender-matched control groups participated in the most recent pilot study by Banerjee and associates. PH patients had much greater amounts of circulating nonfunctional SCN5a splicing variant (SV) mRNA than the control group. Furthermore, they discovered that baseline mPAP and PVR and SCN5a SV expression levels were strongly correlated negatively^92^. Recent findings suggest that higher levels of splicing factors RNA binding motif protein 25 (RBM25) and LUC7 like precursor mRNA splicing factor (LUC7L3), which are induced by hypoxia and/or accelerated angiotensin II mediated signaling, are responsible for heightened levels of SCN5a SV. Both RBM25 and LUC7L3 belong to the RBP family, which is involved in pre-mRNA splicing. They are linked to a number of splicing elements, including U1 snRNP. Splicing utilizing the recessive splicing sequence in the SCN5a terminal exon (exon 28) has been demonstrated to be the cause of SCN5a SV due to the higher amount of RBM25 and LUC7L3 as well as the greater utilization of the recessive 30 splice site in the terminal exon^95^. Even though the exact method by which SCN5a SV controls PH is yet unknown, halting this aberrant mRNA processing may lower the mortality rate of PH because of the correlation between channel decrease and sudden death in PH patients.

Vascular remodeling in pulmonary hypertension is significantly impacted by the increased proliferation of PASMCs. Akira Kurosawa and associates screened 5562 compounds in PASMC of PAH patients at high-throughput to find a possible PAH medication that might slow the growth of PASMC. Smooth muscle cell proliferation and ROS generation are known to be controlled by a number of TFs, including HIF1a, NF-KB, and bromodomain protein 4 (BRD4), via lowering cytoplasmic reactive oxygen species (ROS) and the RNA levels of these proteins. Berberine was discovered to be a chemical that may considerably reduce the growth of PAH-PASMCs. Furthermore, they showed that the anti-pH impact of berberine on PAH PASMCs is mediated by the RBP zinc finger C3H1 domain protein (ZFC3H1)^46^. The authors of this work came to the conclusion that ZFC3H1, a Celastramycin binding partner, controlled nuclear RNA stability to mediate the suppression of HIF1a and BRD4 RNAs. Through persistent pulmonary vasoconstriction, chronic hypoxia also causes pulmonary hypertension^96^. The downregulation of the soluble guanylate cyclase (sGC), which generates nitric oxide, a strong vasodilator, is one of the initial effects of hypoxia exposure^37^. The RBP HuR, which stabilizes its expression, is at least partially responsible for sGC stability. HuR is down-regulated in hypoxia, which leads to prolonged vasoconstriction and a reduction in sGC expression^37^. The involvement of one RBP, HNRNPA2B1, in PAH90 was recently investigated transcriptome-wide for the first time by Ruffenach and associates^51^. They showed that HNRNPA2B1 simultaneously controls thousands of mRNAs in PAH-PASMC. HNRNPA2B1 is a crucial regulator of the PASMC phenotype since the majority of these genes are involved in cell cycle control, including G1/S, DNA synthesis, G2/M, and spindle assembly checkpoints. While HNRNPA2B1 is known to be cytoplasmic and destabilize its targets in cancer, it was demonstrated to be nuclear in PAH and to stabilize its targets^51^. Other investigators showed that human and rodent PH lung and pulmonary artery tissues, as well as hypoxic human PASMCs, had increased levels of mRNA and protein expression of QKI (Quaking), an RNA-binding protein^93^. Vascular remodeling *in vivo* and PASMC proliferation *in vitro* were both reduced by QKI deficiency. Additionally, they demonstrated that QKI binds to the 3' untranslated region of STAT3 (signal transducer and activator of transcription 3) mRNA, increasing its stability. *In vitro*, QKI inhibition decreased PASMC proliferation and STAT3 expression^93^.

Few studies directly examine RBPs in COPD, in contrast to the research of RBPs in PH. Smokers with or without COPD showed elevated HuR expression in their airway epithelium^59^. This implies that smoking is probably the cause of this rise, which is corroborated by another study that found that the bronchial epithelium of smokers without COPD and those with COPD has similar levels of HuR expression^58^. Recent research provides mechanistic evidence that HuR plays a part in the pathophysiology of COPD. For instance, the transcription factor zinc finger E-box binding homeobox 1 (ZEB-1), which is implicated in EMT, is stabilized by HuR. The airway epithelium from COPD had higher ZEB-1 expression^59^. This raises the prospect that HuR regulates EMT and may play a role in the pathophysiology of COPD. Other researchers have not done much study on the function of RBP in COPD; instead, they have mostly concentrated on how it manifests. The bronchial epithelium of COPD patients has lower levels of the RBP ARE/poly(U)-binding/degradation factor 1 (AUF1), which contributes to mRNA decay, than smokers without COPD. Microarray analysis of a primary airway epithelium from COPD showed that AUF1 target genes, particularly those linked to inflammation, are upregulated^58^. Even while this implies that AUF1 could control the expression of inflammatory genes linked to COPD, it is yet unknown if AUF1 directly regulates these downstream mRNAs and what that means for the pathophysiology of COPD. In contrast to smokers and non-smokers, the majority of RBP genes are downregulated in the small airway epithelium of people with COPD, according to a recent mapping profile of RBPs^94^. All things considered, these results suggest that RBPs could play a role in the onset of COPD. While HuR and PTBP1 have distinct roles in COPD and PH, they may share regulatory pathways. For example, both RBPs can be modulated by hypoxia, which is a common pathological driver in both diseases. Hypoxia can upregulate HuR and PTBP1, leading to changes in mRNA stability and splicing that promote disease progression. Therefore, while HuR and PTBP1 have specific roles in COPD and PH, they may also represent convergent molecular mechanisms that are modulated by common pathological stimuli. Besides, RBPs like HuR and PTBP1 could be predictive factors for COPD-PH severity and progression, with their expression levels possibly indicating disease severity, pulmonary artery pressure, and functional capacity. To confirm their prognostic value, future studies should evaluate these RBPs alongside clinical parameters such as spirometric parameters (e.g., FEV1, FVC), pulmonary artery pressure (measured by right heart catheterization or echocardiography), exercise capacity (assessed by 6-minute walk distance or cardiopulmonary exercise testing), and biomarkers of inflammation and remodeling (e.g., TNF-α, IL-1β, NT-proBNP). Longitudinal follow-up is also essential to track disease progression and outcomes. By correlating RBP expression with these parameters, researchers can determine their prognostic value and potential utility in clinical practice.

ZFC3H1 has been shown to mediate the suppression of HIF1α and BRD4 RNAs in PH^46^, but its role in COPD remains unknown. In both COPD and PH, hypoxia plays a significant role in driving disease progression. The hypoxia-driven regulation of RBPs such as HuR and ZFC3H1 has been well described in PH, where these RBPs contribute to vascular remodeling and inflammation. In COPD, hypoxia also contributes to disease progression, but the specific mechanisms involving RBPs are less well understood. However, it is likely that similar hypoxia-driven pathways are involved in both conditions. For example, hypoxia can upregulate HuR expression in COPD, leading to increased stability of mRNAs encoding pro-inflammatory cytokines such as TNF-α and IL-1β, thereby exacerbating inflammation in the airways. Similarly, ZFC3H1 may be involved in regulating the expression of genes related to hypoxia response and vascular remodeling in COPD, although its specific role remains to be elucidated. We propose that future studies could investigate whether ZFC3H1 is dysregulated in COPD and whether it contributes to the development of PH in COPD patients. Additionally, we suggest exploring the role of post-translational modifications of RBPs, such as phosphorylation and ubiquitination, in COPD-PH comorbidity. For instance, the phosphorylation of HuR has been shown to regulate its nuclear-cytoplasmic shuttling and mRNA-binding activity^97^, and this could be a potential mechanism by which HuR contributes to the pathogenesis of COPD and PH.

Based on the current understanding, it is likely that these RBPs operate both independently and synergistically in the progression of COPD and PH. For example, HuR and PTBP1 have distinct roles in regulating mRNA stability and splicing, respectively, but they may also interact in certain contexts. HuR stabilizes mRNAs encoding pro-inflammatory cytokines in COPD, while PTBP1 represses the splicing of the PKM gene, leading to increased production of the PKM2 isoform, which is associated with increased glycolysis and vascular smooth muscle cell proliferation in PH. In some cases, these RBPs may antagonize each other's functions, depending on the specific mRNA targets and cellular context.

Transcriptomic studies have identified both condition-specific and shared gene expression signatures in COPD and PH. For example, the expression of genes related to inflammation, such as TNF-α and IL-1β, is upregulated in COPD, while the expression of genes related to vascular remodeling, such as CD40 and BMPR2, is dysregulated in PH^58^, ^91^. However, there are also shared gene expression signatures between COPD and PH, such as the upregulation of genes related to hypoxia and oxidative stress. These shared signatures could provide the basis for biomarkers or therapeutic targets that are applicable to both diseases. For instance, the RBP HuR has been shown to be upregulated in the airway epithelium of COPD patients and in pulmonary vascular tissues of PH patients^37^, ^59^, suggesting its potential as a biomarker for these diseases. Additionally, the RBP SFPQ has been implicated in the transcriptional regulation of genes involved in vascular remodeling in PH^38^, and its expression could serve as a prognostic biomarker for disease progression. The choice of RBPs for clinical application should be based on several factors, including their specificity for the disease, their accessibility for measurement (e.g., in blood or sputum), and their association with disease outcomes. Future studies could validate the potential of these RBPs as biomarkers through large-scale clinical trials and explore their utility in guiding therapeutic decisions. Additionally, targeting shared pathways, such as the hypoxia response pathway, could provide therapeutic benefits for both diseases.

To date, few studies have specifically investigated unique alternative splicing events associated with COPD-PH comorbidity. However, RNA-seq and alternative splicing analysis could provide valuable insights into the role of RBPs in this context. By comparing the transcriptomes of patients with COPD, PH, and COPD-PH comorbidity, researchers could identify distinct splicing events regulated by RBPs that contribute to the pathogenesis of these diseases. This approach could help identify novel therapeutic targets and biomarkers specific to COPD-PH comorbidity. Genetic polymorphisms in RBPs, such as SNPs, have been associated with altered RBP function and disease susceptibility. Additionally, epigenetic modifications, such as DNA methylation and histone modification, can regulate the expression and activity of RBPs. For example, the expression of HuR can be modulated by DNA methylation^98^, and this may contribute to its dysregulation in COPD and PH. Future studies could investigate whether specific SNPs or epigenetic changes in RBPs are associated with altered disease risk, severity, or therapeutic response in COPD-PH comorbidity^99^-^102^. This could involve genotyping and epigenetic profiling of patient cohorts, as well as functional studies to assess the impact of these variations on RBP activity and downstream gene expression.

### Interplay of RBPs with ncRNAs in PH and COPD

Understanding the functional significance of RBPs and ncRNAs in the development of PH and COPD may be aided by the knowledge that ncRNAs interact with RBPs. As demonstrated by studies in some cancer cells and more recently in vascular smooth muscle cells, miR-124's proximal direct target, the RNA-binding protein PTBP1, is a crucial regulator of the effects of miR-124 on PH fibroblast proliferation and migration^90^. MCF2L-AS1 interacts with the CCND1 mRNA to increase its stability after transcription factor E2F1 binds to the promoter region in association with HuR/ELAVL1^103^. Higher levels of miR-4640-5p was associated with more severe COPD-PH, and it was considerably greater in the lung tissues of COPD-PH patients than in healthy controls. Potentially controlled by AUF1, overexpression of NOS1 partially counteracted the impact of miR-4640-5p in controlling the migration and proliferation of PASMC cells^33^. Additionally, the RBP SRSF2 has the ability to control the BMPR2 gene's splicing, which miR-126 targets in pulmonary arterial hypertension^91^. RBPs and ncRNAs have been found to interact, which emphasizes how they control the post-transcriptional processes of target mRNAs. This would be an additional step toward comprehending the gene landscape implicated in the pathophysiology of COPD and PH.

### Clinical significance of RBP-regulating mRNA translation and stability as therapeutic targets in PH and COPD

As previously indicated, the pathophysiology of PH and COPD may be impacted by the translation-associated RBPs and mRNA stability. Drugs that target the same RBP in cancer may provide us some clues, even if there aren't any that specifically target COPD and PH-related RBPs in clinical practice at the moment. HuR is a typical RBP that controls the stability of mRNA and exhibits abnormal expression in a variety of cancer types. Following decades of research, a group of HuR inhibitors that block the RNA-binding domain of RRM1 and RRM2 have been created. These inhibitors include MS-444, okicenone, dehydromutactin, DHTS, and AZA-9, and they have demonstrated a nanomolar inhibitory impact on HuR^97^, ^104^-^106^. HuR inhibitors block the RNA-binding domain of HuR, preventing its interaction with mRNAs containing AU-rich elements (AREs) in their 3' untranslated regions (3'UTRs). This leads to the destabilization and degradation of these mRNAs, reducing the expression of pro-inflammatory cytokines and other proteins involved in disease progression. These inhibitors caused HuR to lose its ability to bind to ARE-containing mRNA targets, including IL-2, IL-1β, TNF-α, COX-2, and c-fos. As a result, HuR-mediated stability was no longer protected, and colorectal cancer displayed specific anti-cancer effects^106^. Recently, CMLD-2, another powerful inhibitor of HuR, was created to treat thyroid cancer and non-small-cell lung carcinoma. It competitively binds to HuR and suppresses the expression of Mad2, Bax, and Bcl-xL, which enhances tumor cell death and prevents the growth of cancer^107^, ^108^. One of the major challenges in using HuR inhibitors is the potential for off-target effects. Since HuR is involved in the regulation of a wide range of mRNAs, non-specific binding of inhibitors to other RNA-binding proteins or cellular components could lead to unintended consequences. For example, the inhibition of HuR could affect the stability of mRNAs involved in normal cellular processes, leading to disruptions in cell function and viability. HuR is involved in the regulation of immune responses, and its inhibition could potentially lead to unintended immune-related side effects. For instance, the reduction in the expression of pro-inflammatory cytokines could dampen the immune response, making patients more susceptible to infections. The delivery of HuR inhibitors to the target tissues, particularly the lungs, is another challenge. The inhibitors need to be efficiently delivered to the pulmonary vasculature and airway epithelium to exert their therapeutic effects. Nanoparticle-based carriers or inhalable formulations could be developed to enhance the targeting and reduce off-target effects.

Because of its increased translational control over carcinogenic genes, eIF4E, the central component of the 5' cap-dependent translation initiation complex, is overexpressed in a variety of malignancies and facilitates neoplastic transformation. Decades of work have gone into creating eIF4E inhibitors since it is believed that carcinogenic mRNAs have the greatest eIF4E demand for translation in the genesis of cancer. Preclinical studies have demonstrated that eIF4E inhibitors can effectively reduce the growth and proliferation of cancer cells. For example, Ribavirin, an antiviral guanosine analog, was initially discovered to physically mimic the m7G cap-structure and competitively inhibit eIF4E's association with a group of oncogenes, including CyclinD1, lowering their translation efficiency and therefore stopping tumorigenesis^109^. Recently, a different drug called 4Ei-1 was created to inhibit eIF4E-cap binding and cause eIF4E to be broken down by proteases. This causes breast cancer and lung cancer cells to become more sensitive to gemcitabine therapy in a synergistic manner, suggesting its potential as a therapeutic agent^110^. In addition to the antagonist binding approach, 4EGI-1 and 4E1RCat protein-protein interaction (PPI) inhibitors were screened out in order to interfere with eIF4E's affinity for its cofactors, eIF4G and eIF4A. This disrupts the cap-dependent translation machinery and causes the pro-apoptotic effects that are unique to cancer cells^111^, ^112^. eIF4E is involved in the translation of a wide range of mRNAs, and its inhibition could lead to off-target effects. Non-specific binding of inhibitors to other translation initiation factors or cellular components could disrupt normal cellular processes, leading to unintended consequences. The systemic administration of eIF4E inhibitors could lead to toxicity in non-target tissues, particularly in the liver and kidneys. This could limit the therapeutic potential of these inhibitors and require careful dose optimization. Cancer cells may develop resistance to eIF4E inhibitors over time, limiting their long-term efficacy. This could be due to the upregulation of alternative translation initiation pathways or the acquisition of mutations that render the inhibitors ineffective.

Targeting RBPs such as HuR, and eIF4E holds significant therapeutic potential for the treatment of COPD and PH. However, the feasibility and potential side effects of these inhibitors need to be carefully evaluated. A valid concern is the potential for widespread inhibition of critical RBPs to disrupt normal cellular processes. Since RBPs play essential roles in immune regulation, wound healing, and tissue repair, their inhibition could lead to unintended consequences. For example, inhibiting RBPs involved in the stability of mRNAs encoding pro-inflammatory cytokines could dampen the immune response, increasing susceptibility to infections. Similarly, inhibiting RBPs that regulate the expression of genes involved in tissue repair could impair wound healing and regeneration. To minimize these risks, future research should focus on several key strategies. First, developing highly specific inhibitors that target only the disease-related functions of RBPs while sparing their normal cellular functions is crucial. Second, using targeted delivery systems such as nanoparticles or inhalable formulations can help deliver RBP inhibitors specifically to the affected tissues, such as the pulmonary vasculature or airway epithelium, thereby reducing systemic exposure. Third, comprehensive preclinical studies should be conducted to evaluate the safety and efficacy of RBP inhibitors, with a particular focus on immune function and tissue homeostasis. Lastly, exploring combination therapies that target multiple RBPs or pathways involved in disease progression could potentially allow for lower doses of individual inhibitors, thereby reducing off-target effects.

## Conclusions

RBPs are dysregulated in PH and COPD, according to new *in vitro* and *in vivo* experimental evidence, and they significantly impact the phenotypic of pulmonary vascular cells as well as the pathogenesis of the illness as a whole. Because of their intricate biology, which is greatly influenced by their post-translational and epitranscriptomic modifications, RBPs can quickly adapt to both pathologic and homeostatic cell conditions. They can also have a significant functional impact by coordinating the regulation of multiple gene sets and modifying their protein output to meet the demands of the microenvironment. RBPs may be especially useful as therapeutic targets because of their involvement in inflammation and hypoxia, which have been previously identified in preclinical and clinical models of PH and COPD. To further understand RBPs' role in responses shared by PH and COPD, as well as their therapeutic targetability, more preclinical research and specialized mice models are obviously required.

Before we fully comprehend the extent of RBP regulation, we still have a lot to learn about how RBPs act in COPD and PH. We suggest that future studies should focus on identifying RBP-specific targets for individualized therapy in COPD and PH. This could involve the development of biomarkers based on the expression or activity of specific RBPs, such as HuR or SFPQ, to stratify patients and guide targeted therapies. Additionally, we propose that future research should investigate the role of RBPs in early disease stages, as this could provide insights into the mechanisms of disease initiation and progression. For instance, studies could explore whether dysregulation of RBPs such as QKI, which has been shown to promote vascular remodeling in PH, occurs early in the development of COPD and PH. Besides, existing preclinical models may not fully capture the complexity of RBP dynamics *in vivo*. To more accurately recreate the *in vivo* disease-promoting microenvironment, we suggest improvements to existing models, such as the use of genetically modified animals that specifically target RBPs or their downstream targets. Additionally, we propose the development of new experimental models, such as humanized mouse models or organoid systems, which could better mimic the human disease environment and allow for more detailed studies of RBP dynamics. The integration of bioinformatics, high-throughput molecular biology approaches, and nanotechnology could provide a comprehensive approach to identifying and targeting RBPs in these diseases^113^-^115^. For example, bioinformatics tools could be used to predict the binding sites of RBPs on mRNAs and identify potential therapeutic targets. High-throughput molecular biology approaches, such as RNA sequencing and proteomics, could be used to validate the predicted targets and quantify the impact of RBPs on gene expression. Nanotechnology could be used to develop efficient delivery systems for RBP-targeted therapies, such as nanoparticle-based carriers that enhance the targeting of drugs to pulmonary tissues^100^, ^116^, ^117^. Given the heterogeneity of COPD, it is plausible that RBP-related mechanisms may vary between different phenotypes such as emphysema-dominant and chronic bronchitis-dominant subtypes. For example, the expression and activity of RBPs such as HuR and AUF1 may differ between these subtypes, reflecting the distinct pathophysiological processes involved. Emphysema-dominant COPD may involve more pronounced oxidative stress and extracellular matrix remodeling, potentially affecting RBP regulation differently compared to chronic bronchitis-dominant COPD, which is characterized by airway inflammation and mucus hypersecretion. Future studies could investigate RBP expression profiles in different COPD phenotypes to identify subtype-specific mechanisms and therapeutic targets. The combination of these interdisciplinary strategies could accelerate the discovery and development of RBP-targeted therapies for COPD and PH, ultimately leading to improved therapeutic outcomes for patients.

## Author contributions

YL, TJ and RW wrote the manuscript, and prepared the figures and tables; YL conceived and revised the manuscript. All authors read and approved the manuscript in its final form.

## Figures and Tables

**Table 1:** Role of RBPs in PH and COPD.

RNA-Binding Protein	Targets	Effect on target mRNA	Functional Implication	Reference
SFPQ	CD40	Represses transcription	By triggering CD40 transcription, decreased RBP SFPQ levels encourage the activation of pulmonary artery adventitial fibroblasts.	38
PTBP1	PKM (PKM1 and PKM2)	Represses splicing	Elevated splicing repressor PTBP1 levels prevent exon 9 of PKM pre-mRNA from being used, which increases PKM2 production and modifies the phenotypes of PH circulating and vascular cells.	39, 90
SRSF2	BMPR2 (isoform B and A)	Activates splicing	The decreased penetrance among BMPR2 heterozygous mutant carriers can be explained by the fact that decreased amounts of the splicing activator SRSF2 raise the levels of non-functional BMPR2 B isoform in PH cells from afflicted BMPR2 mutation carriers (who develop PH).	91
HF related splicing factor(s)	SCN5a	Activates splicing?	In heart failure and PH, elevated RBP levels (which may be caused by RBM25 and LUC7L3) encourage the development of a non-functional splicing variant of SCN5a.	92
ZFC3H1	BRD4 and HIF1	Stabilizes mRNA	By controlling the breakdown of nuclear RNAs in PASMCs, ZFC3H1 functions as a binding partner of Celastramycin and mediates the suppression of BRD4 and HIF-1a by Celastramycin therapy.	46
HuR	sGC-1 ZEB-1?	Stabilizes mRNA	In rat pulmonary arteries, increased HuR protein translocation from the cytoplasm to the nucleus destabilizes sGC-a1 mRNA, lowering NO/sGC signaling in response to transient hypoxia. HuR stabilizes zinc finger E-box binding homeobox 1 (ZEB-1), a transcription factor linked to EMT in COPD.	37, 58, 59
HNRNPA2B1	Cell Cycle Checkpoints	Stabilizes mRNA	HNRNPA2B1 stimulates the expression of its targets in PAH-PASMC. All evaluated mRNAs with an HNRNPA2B1 motif decreased as a result of HNRNPA2B1 silencing in PAH-PASMC, which also caused a decrease in proliferation and apoptosis resistance.	51
QKI	STAT3	Stabilizes mRNA	Human and rodent PH lung and pulmonary artery tissues, as well as hypoxic human PASMCs, showed increased levels of QKI mRNA and protein expression. Vascular remodeling *in vivo* and PASMC proliferation *in vitro* were both reduced by QKI deficiency. QKI binds to the 3' untranslated region of STAT3 mRNA, increasing its stability.	93
AUF1	NR	NR	Compared to smokers without COPD, the bronchial epithelium of COPD patients has reduced levels of the RBP AUF1, which aids in mRNA degradation.	58, 94
